# Greater valgus severity is associated with larger lateral flexion‐gap changes between intraoperative assessments during robot‐assisted total knee arthroplasty: A retrospective cohort study

**DOI:** 10.1002/jeo2.70976

**Published:** 2026-11-03

**Authors:** Yugo Morita, Jonggu Shin, Mohammed El‐Hassan, Ittai Shichman, Skylar Lewis, John M. Dundon, William J. Long, Peter K. Sculco

**Affiliations:** ^1^ Complex Joint Reconstruction Center Adult Reconstruction and Joint Replacement Service, Hospital for Special Surgery New York New York USA; ^2^ Orthopedic Research Institute of New Jersey Chester New Jersey USA; ^3^ Atlantic Health System Morristown New Jersey USA

**Keywords:** coronal alignment, flexion gap, robot‐assisted total knee arthroplasty, soft‐tissue gap balance, valgus knee

## Abstract

**Purpose:**

To characterize differences between intraoperative flexion‐gap assessments and their association with deformity severity during primary robot‐assisted total knee arthroplasty for osteoarthritis.

**Methods:**

This single‐centre retrospective cohort included 512 eligible knees identified from an institutional database between August 2022 and July 2025. One surgeon used different alignment strategies for varus and valgus knees. Robot‐recorded gaps were assessed at 90° using manual varus–valgus stress before bone resection and a quantitative tensioner after distal femoral and proximal tibial resections, extension balancing and selective releases when required. The primary outcome was compartment‐specific gap change: post‐resection measurements minus tibial resection depths and initial gaps. Multivariable models examined associations with deformity severity and clinical outcomes.

**Results:**

Paired measurements were available for 505 knees (438 patients); 465 had final measurements. Tibial‐resection‐adjusted gaps exceeded initial gaps by a mean of 1.5 mm medially (95% confidence interval [CI] 1.2–1.7) and 0.8 mm laterally (95% CI 0.5–1.1; both *p* < 0.001). In 139 valgus knees, each additional degree of valgus was associated with 0.24‐mm greater lateral gap change (95% CI 0.14–0.34; Holm‐adjusted *p* < 0.001). Using an absolute mediolateral difference ≤2.5 mm to define balance, 21/42 (50.0%) initially balanced valgus knees were reclassified as imbalanced, 20 with larger lateral gaps; 16/21 (76.2%) were balanced at final assessment. Reclassification rates were 58.3% and 44.4% with 2‐ and 3‐mm thresholds. Medial, lateral and mean compartment‐specific gap changes were not associated with the 1‐year Knee injury and Osteoarthritis Outcome Score for Joint Replacement in 342 knees (all Holm‐adjusted *p* = 0.959).

**Conclusion:**

Flexion‐gap measurements differed between intraoperative checkpoints, with greater valgus severity associated with larger lateral opening. Initial balance did not consistently persist at the later checkpoint, demonstrating that the information available for femoral planning may differ from that obtained during the initial manual‐stress assessment.

**Level of Evidence:**

Level III, retrospective cohort study.

**Abbreviations**
BMI: body mass index
CI: confidence interval
CR: cruciate‐retaining
HC1: heteroscedasticity‐consistent Type 1
HKA: hip–knee–ankle angle
IQR: interquartile range
IRB: institutional review board
JLCA: joint‐line convergence angle
KOOS‐JR: Knee injury and Osteoarthritis Outcome Score for Joint Replacement
LDFA: lateral distal femoral angle
L‐FKP: laxity‐functional knee phenotype
MPTA: medial proximal tibial angle
OR: odds ratio
PCA: posterior condylar axis
PCL: posterior cruciate ligament
PS: posterior‐stabilized
Q1: first quartile
Q3: third quartile
REAL: Robotic Evaluation of Articular Laxity
ROSA: Robotic Surgical Assistant
TKA: total knee arthroplasty

## INTRODUCTION

Flexion‐gap assessment is an important component of femoral planning and soft‐tissue balancing in robot‐assisted total knee arthroplasty (TKA). Robotic systems incorporate pre‐resection compartment gaps into component planning, but measurements depend on how the tissues are tensioned [3]. Manual varus–valgus stress is widely used but is not standardized and may vary with surgeon experience [17, 27], whereas instrumented tensioners provide controlled distraction [4, 30]. Bone resection and sequential balancing can also alter flexion gaps, with changes persisting after adjustment for resection thickness [1, 20, 31]. Consequently, the initial gap pattern may differ from that encountered later during surgery.

These differences may be particularly relevant in knees with coronal deformity. Valgus severity has been associated with extension‐gap asymmetry but not consistently with flexion‐gap asymmetry, indicating substantial variation in the flexion soft‐tissue envelope [6]. Laxity‐functional knee phenotype (L‐FKP) studies similarly show that osseous alignment does not determine a uniform flexion‐laxity pattern [8, 9]. Personalized alignment, deformity correction and selective soft‐tissue procedures may further modify gap balance [5, 7, 10, 15, 16]. However, the magnitude and direction of within‐knee changes between initial assessment and subsequent femoral planning remain incompletely characterized, as do their relationship with deformity severity and the frequency of balance reclassification.

The subsequent flexion assessment is obtained while femoral rotation and posterior condylar resection remain adjustable, making differences between assessments relevant to femoral planning. Accordingly, this retrospective study evaluated patients undergoing primary robot‐assisted TKA for osteoarthritis to quantify within‐knee compartment‐specific gap changes between pre‐resection manual varus–valgus stress and quantitative tensioning after distal femoral and proximal tibial resections and extension balancing.

The primary endpoint was compartment‐specific gap change, defined as the tibial‐resection‐adjusted rotation‐tool gap minus the initial manual‐stress gap, evaluated separately medially and laterally. Secondary analyses examined alignment, deformity severity, compartment‐specific opening, balance reclassification and 1‐year patient‐reported outcomes. Associations between changes in mediolateral gap difference and final imbalance were exploratory. The primary hypothesis was that flexion‐gap measurements would differ between assessments; the secondary hypothesis was that greater varus and valgus severity would be associated with larger medial and lateral gap changes, respectively.

## MATERIALS AND METHODS

### Study design and cohort

The institutional database was reviewed for all consecutive primary robot‐assisted TKAs for osteoarthritis performed by one surgeon between August 2022 and July 2025. Of 534 knees screened, 22 were excluded for rotating‐hinge implants (*n* = 7), post‐traumatic deformity (*n* = 12) or unavailable preoperative radiographs (*n* = 3), leaving 512 eligible knees in 442 patients. Paired initial manual‐stress and post‐resection rotation‐tool measurements were available for 505 knees in 438 patients, forming the primary cohort. Forty lacked complete final flexion‐gap measurements, leaving 465 for three‐assessment analyses (Figure 1). Sample size was determined by all eligible cases available during the predefined period rather than prospective recruitment to a numerical target.

**Figure 1:**
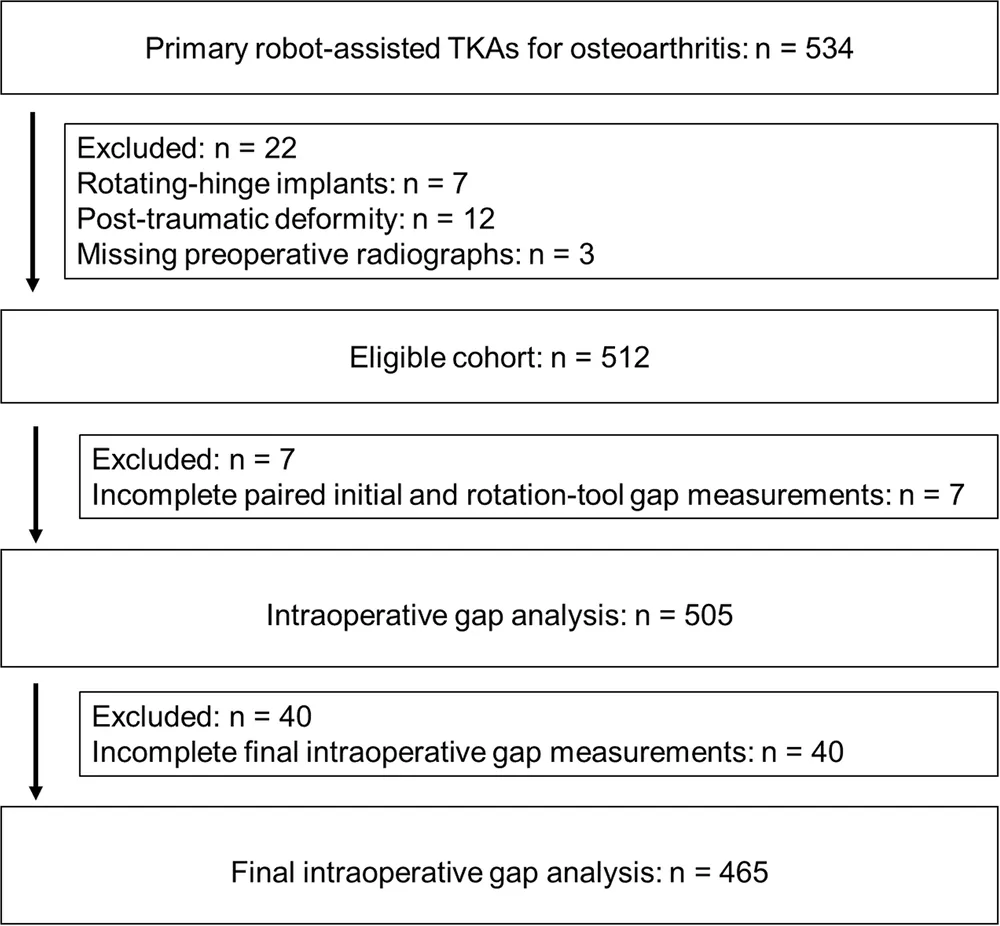
Flow diagram of knee selection for the intraoperative gap analyses. Among 534 primary robot‐assisted TKAs performed for osteoarthritis, 22 knees were excluded because of rotating‐hinge implants, post‐traumatic deformity or unavailable preoperative radiographs. Of the remaining 512 eligible knees, seven were excluded because paired initial manual‐stress and post‐resection rotation‐tool measurements were unavailable, leaving 505 knees for the intraoperative gap analysis. A further 40 knees were excluded because of incomplete final trial‐component gap measurements, leaving 465 knees for the final intraoperative gap analysis. TKA, total knee arthroplasty.

### Surgical technique and intraoperative gap measurements

All procedures used a standard medial parapatellar approach and the imageless Robotic Surgical Assistant (ROSA) Knee System (Zimmer Biomet) [21]. Femoral and tibial trackers were fixed with percutaneous pins, and landmarks were registered according to the manufacturer's workflow [2]. Before resection, the same surgeon applied manual varus–valgus stress in extension and at 90° of flexion while the robot recorded compartment gaps and maximum varus and valgus angles. Each assessment was acquired once per position without force‐controlled tensioning or repeated acquisitions for reliability testing. Hip rotation facilitated endpoint identification during flexion assessment. When substantial internal rotation obscured the endpoint, the knee was supported against the surgeon's chest while manual valgus stress was applied to the foot. This was a positioning modification within the same manual‐stress assessment; its use was not recorded per case. Pre‐resection flexion spaces were termed initial manual‐stress gaps. Initial flexion laxity range was calculated as maximum varus minus maximum valgus angle.

Varus knees underwent functional alignment, with component positioning guided by compartment gaps within predefined boundaries [5, 7, 15]. Intended coronal orientation was approximately within 3° of tibial varus, 2° of femoral valgus to 1° of varus and overall hip–knee–ankle angle (HKA) from 2° of valgus to 3° of varus. Mild additional residual varus was accepted in selected constitutionally varus knees when compatible with the gap and laxity profile, although HKA > 4° of varus was generally avoided.

Valgus knees underwent a femoral‐referenced strategy with a slight valgus mechanical‐alignment target [10, 15]. Intended postoperative HKA ranged from neutral to approximately 1.5° of residual valgus when compatible with the soft‐tissue envelope. Tibial coronal resection was neutral, and femoral orientation was generally 1° of valgus, with up to 2° permitted. Tibial slope followed native anatomy, typically between 5° and 8°. The displayed extension‐compartment value combined planned distal femoral and proximal tibial resection depths with measured laxity. Relative to a 19.0‐mm implant construct, targets were 20.0–20.5 mm laterally in varus knees and 18.0–18.5 mm medially in valgus knees, corresponding to theoretical residual gaps of 1.0–1.5 mm and −1.0 to −0.5 mm, respectively. In valgus knees requiring release, this was performed in extension after meniscal removal, beginning with the posterolateral corner and followed by graded iliotibial band pie‐crusting if necessary [26]. Varus knees underwent osteophyte removal and, rarely, tibial reduction osteotomy without ligament release.

After the initial assessment, distal femoral and proximal tibial resections were performed and compartment‐specific depths were validated robotically. Extension balance was reassessed using spacer blocks, with additional resection‐level adjustment and the selective procedures described above when required. At 90° of flexion, quantitative tensioning was performed using a Physica Knee Balancer (Enovis). Both paddles were advanced to equal nominal target‐force settings, typically approximately 100 N per compartment in women and 120 N in men, following the surgeon's standard protocol. The femur was manually elevated, and ROSA recorded the raw compartment‐specific distraction distances. This post‐resection rotation‐tool assessment preceded and guided final femoral rotation and posterior condylar resection (Figure 2).

**Figure 2:**
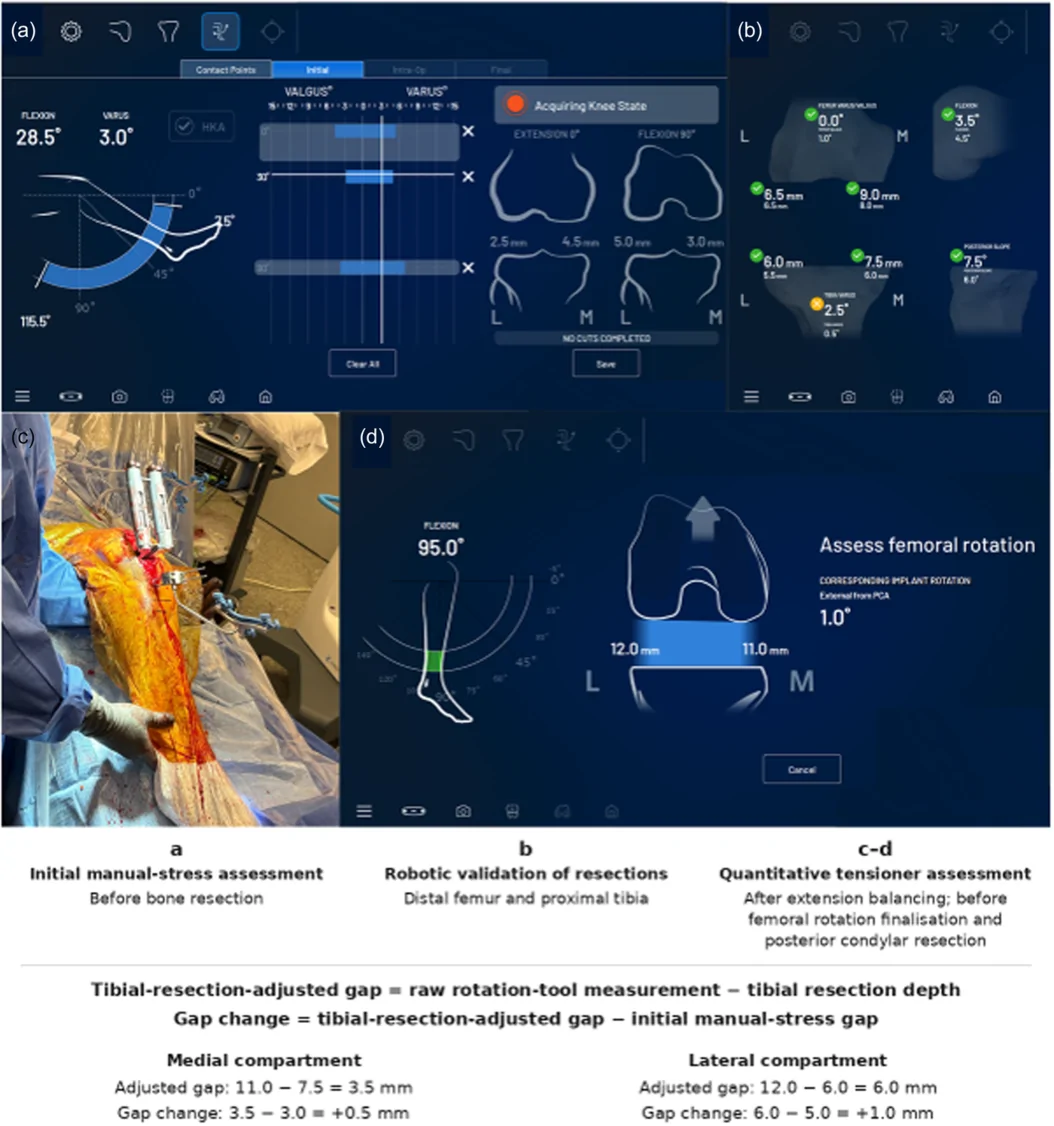
Intraoperative sequence of flexion‐gap assessment and tibial‐resection adjustment. The initial assessment was obtained using surgeon‐applied manual stress before bone resection (a). Panel (b) shows robotic validation of distal femoral and proximal tibial resections. After extension balancing, quantitative tensioning was performed before finalization of femoral rotation and posterior condylar resection (c), and ROSA recorded the raw compartment‐specific distraction distances (d). The raw medial and lateral measurements were adjusted by subtracting the corresponding compartment‐specific proximal tibial resection depth; worked examples and changes from the initial assessment are shown in the figure. HKA, hip–knee–ankle angle; L, lateral; M, medial; PCA, posterior condylar axis; ROSA, Robotic Surgical Assistant.

Raw rotation‐tool distances were referenced to the resected tibial level. Following the resection‐based reference framework previously used in this workflow [20], robot‐validated compartment‐specific proximal tibial resection depths were subtracted to express measurements relative to the pre‐resection tibial reference. These values were termed tibial‐resection‐adjusted rotation‐tool gaps. The correction accounted for the geometric reference change without recreating the initial biomechanical state. Compartment‐specific gap change was the adjusted gap minus the initial manual‐stress gap; positive values indicated opening. In posterior‐stabilized (PS) knees, the posterior cruciate ligament (PCL) was resected during proximal tibial resection, between the initial and rotation‐tool assessments. In cruciate‐retaining (CR) knees, it was preserved at this stage.

After femoral preparation, posterior osteophytes were removed, and limited posterior capsular release was performed when necessary. With trial components in place, flexion gaps were reassessed at 90° using the same manual‐stress technique and recorded as final trial‐component gaps [22]. Complete PCL release was not performed in CR knees. Persistent tibial trial lift‐off despite component adjustment was treated with limited anterolateral PCL fibre release sufficient to eliminate lift‐off, followed by reassessment of gaps and varus–valgus laxity.

### Assessment terminology

The three checkpoints are referred to as initial manual‐stress, post‐resection rotation‐tool and final trial‐component assessments. Initial and post‐resection balance classifications used the initial gaps and tibial‐resection‐adjusted rotation‐tool gaps, respectively. Initial‐to‐post‐resection transitions therefore preceded final femoral rotation and posterior condylar resection. Subsequent femoral preparation, trialling and final balancing were reflected only in the final assessment.

### Radiographic measurements and phenotype definitions

Standing full‐length weight‐bearing radiographs were obtained preoperatively. The hip–knee–ankle (HKA) angle was measured between the femoral mechanical axis, connecting the centres of the femoral head and knee and the tibial mechanical axis, connecting the centres of the knee and ankle. Varus alignment was recorded as positive and valgus alignment as negative. Knees were classified using the preoperative HKA as varus (≥3°), neutral (>−3° and <3°) or valgus (≤−3°) [19]. Among varus and valgus knees, coronal deformity severity was expressed as a positive value and defined as the absolute value of the preoperative HKA angle.

### Statistical analysis

Continuous variables were summarized as medians [Q1–Q3] and categorical variables as *n* (%). Linear effects were reported as mean differences or *β* coefficients and logistic effects as odds ratios (ORs), with 95% confidence intervals (CIs). Regression analyses used patient‐level cluster‐robust HC1 standard errors to account for bilateral knees. Primary paired mean gap changes were estimated using intercept‐only linear models with patient‐level cluster‐robust HC1 standard errors and *t*‐based inference.

Alignment‐group differences in tibial‐resection‐adjusted rotation‐tool gaps were evaluated using analysis of covariance adjusted for the corresponding initial gap, initial flexion laxity range, age, sex and body mass index (BMI). Within varus and valgus groups, linear models related continuous deformity severity to the adjusted rotation‐tool gap using the same covariates. Because initial gap was included, the severity coefficient represented its association with compartment‐specific gap change. Opening of ≥2 mm was evaluated using logistic regression with the same covariates. Medial and lateral compartments were designated as deformity‐corresponding in varus and valgus knees, respectively.

The signed mediolateral difference was calculated as lateral minus medial gap. For directional analyses, its sign was reversed in varus knees so that positive between‐assessment changes indicated shifts towards a larger medial gap in varus knees and a larger lateral gap in valgus knees. Post‐resection minus initial directional differences were modelled against severity, adjusting for initial directional difference, initial flexion laxity range, age, sex and BMI. In valgus knees, probabilities of ≥2‐mm lateral opening were standardized over the model cohort's covariate distribution across the observed severity range and at 5°, 10° and 15°.

Balance was defined as an absolute mediolateral difference ≤2.5 mm using a 2.5‐mm boundary informed by the Robotic Evaluation of Articular Laxity (REAL) framework [14, 19]. Larger differences were classified as imbalance with a larger lateral or medial gap. The ≥2‐mm opening threshold addressed a separate construct: change within one compartment between assessments, rather than mediolateral asymmetry at one assessment. Balance distributions and initial‐to‐post‐resection transitions were summarized overall and by alignment group. Changes in proportions were evaluated using 2000 patient‐level bootstrap samples. Sensitivity analyses repeated balance‐transition analyses using 2‐ and 3‐mm thresholds.

Absolute between‐assessment change in mediolateral difference was the absolute value of post‐resection minus initial lateral‐minus‐medial differences. Exploratory linear and logistic models related this change to final absolute mediolateral difference and final imbalance (>2.5 mm), respectively, adjusting for initial absolute mediolateral difference, initial flexion laxity range and preoperative HKA. Analyses were repeated within alignment groups and with >2‐ and >3‐mm final‐imbalance thresholds.

The Knee injury and Osteoarthritis Outcome Score for Joint Replacement (KOOS‐JR) was assessed preoperatively and at 1 year postoperatively. Postoperative scores were collected by telephone or during outpatient visits; missing postoperative scores reflected non‐response. Associations of medial, lateral and mean compartment‐specific gap changes with 1‐year postoperative KOOS‐JR were evaluated using separate linear models adjusted for preoperative KOOS‐JR, age, sex, BMI and preoperative HKA. The mean gap change was calculated as the average of the medial and lateral compartment‐specific changes. KOOS‐JR analyses were considered secondary, and Holm correction was applied across the three gap‐change exposures.

Final implant type was excluded from primary covariates because it was selected intraoperatively according to ligament integrity, stability and balancing requirements. Sensitivity analyses additionally adjusted for implant type and repeated severity analyses in CR knees and women only. The latter addressed sex‐based tensioner settings. Opening analyses were repeated using ≥1‐ and ≥3‐mm thresholds. To assess dependence on tibial‐resection subtraction, raw rotation‐tool distance was also modelled with the corresponding resection depth as an estimated covariate.

Holm correction was applied across medial and lateral primary comparisons and overall alignment tests, pairwise alignment contrasts within compartments, medial and lateral severity associations within each alignment group and outcome, the two directional‐severity tests and the three KOOS‐JR exposures. Exploratory final‐outcome analyses were also multiplicity‐adjusted, including correction across three alignment groups for each outcome. Alternative thresholds, restricted subgroups, implant adjustment and resection‐depth models were interpreted as robustness analyses rather than independent confirmatory tests.

Analyses used available paired measurements and model‐specific complete cases without imputation. Knees with and without paired gaps, final gaps or paired KOOS‐JR measurements were compared descriptively, using absolute standardized differences where appropriate. Tests were two‐sided with *p* < 0.05, using R version 4.4.2.

## RESULTS

### Patient and cohort characteristics

Of 534 knees screened, 512 knees from 442 patients were eligible, comprising 312 varus, 61 neutral and 139 valgus knees (Table 1). Paired measurements were available for 505 knees from 438 patients. The seven without paired measurements (six varus, one neutral) were descriptively older and had lower BMI and initial flexion laxity. Among paired knees, 40 lacked final measurements. Demographics and initial gaps were broadly similar to those with final measurements, although between‐assessment gap changes were numerically larger (Supporting Information S1: Table 2). Median age in the eligible cohort was 70.0 years [64.0–76.0], 62.5% were female, and median BMI was 31.1 kg/m^2^ [27.1–34.3]. CR and PS implants accounted for 89.1% and 10.9%, respectively.

**Table 1:** Patient and surgical characteristics according to preoperative alignment.

Variable	Overall (*n* = 512)	Varus (*n* = 312)	Neutral (*n* = 61)	Valgus (*n* = 139)
Female sex, *n* (%)	320 (62.5)	170 (54.5)	39 (63.9)	111 (79.9)
Age, years	70.0 [64.0–76.0]	70.0 [64.0–75.0]	70.0 [64.0–75.0]	73.0 [65.0–78.0]
BMI, kg/m^2^	31.1 [27.1–34.3]	31.6 [28.3–35.4]	29.5 [26.9–32.6]	29.4 [25.4–33.1]
Preoperative HKA angle (°)	6.0 [−3.7 to 10.5]	9.4 [7.0–12.8]	0.1 [−1.2 to 1.7]	−8.1 [−10.4 to −5.3]
Preoperative MPTA (°)	86.8 [84.4–89.2]	85.0 [83.5–86.9]	87.6 [86.1–89.0]	90.4 [88.5–92.0]
Preoperative LDFA (°)	87.8 [85.6–90.2]	89.6 [87.9–91.2]	86.7 [85.7–87.7]	84.6 [83.2–85.8]
Preoperative JLCA (°)	3.6 [−0.7 to 5.8]	5.4 [4.0–7.0]	1.2 [−0.5 to 2.4]	−2.4 [−4.2 to −1.0]
Initial flexion laxity range (°)	10.4 [3.3–14.4]	8.9 [2.5–13.1]	9.8 [2.6–15.1]	12.8 [8.6–15.8]
Preoperative KOOS‐JR (points)	52.5 [42.3–59.4]	52.5 [42.3–61.6]	53.7 [42.9–59.4]	50.0 [39.6–59.4]
Implant type, *n* (%)				
CR	456 (89.1)	278 (89.1)	55 (90.2)	123 (88.5)
PS	56 (10.9)	34 (10.9)	6 (9.8)	16 (11.5)

*Note*: Values are median [Q1–Q3] or *n* (%), reported by knee. The eligible cohort comprised 512 knees in 442 patients. Preoperative KOOS‐JR scores were available for 470 knees: 287 varus, 54 neutral and 129 valgus knees. Percentages were calculated using the number of knees with available data. Positive HKA values indicate varus and negative values indicate valgus. Alignment was classified as varus (HKA ≥ 3°), neutral (−3° < HKA < 3°) or valgus (HKA ≤−3°). Patients undergoing bilateral procedures could contribute knees to different alignment groups.

Abbreviations: BMI, body mass index; CR, cruciate‐retaining; HKA, hip–knee–ankle angle; JLCA, joint‐line convergence angle; KOOS‐JR, Knee injury and Osteoarthritis Outcome Score for Joint Replacement; LDFA, lateral distal femoral angle; MPTA, medial proximal tibial angle; PS, posterior‐stabilized; Q1, first quartile; Q3, third quartile.

### Primary compartment‐specific gap change

Tibial‐resection‐adjusted gaps exceeded initial gaps by a mean of 1.5 mm medially (95% CI 1.2–1.7) and 0.8 mm laterally (95% CI 0.5–1.1; both Holm‐adjusted *p* < 0.001). Descriptive gap measurements are presented in Table 2. An overall alignment‐group difference was identified for the adjusted lateral gap (Holm‐adjusted *p* < 0.001), but not for the adjusted medial gap (Holm‐adjusted *p* = 0.176) (Table 3; Figure 3). The adjusted lateral gap was 1.8 mm larger in valgus than in varus knees (95% CI 1.1–2.4; Holm‐adjusted *p* < 0.001) and 1.4 mm larger in valgus than in neutral knees (95% CI 0.5–2.2; Holm‐adjusted *p* = 0.003).

**Table 2:** Initial and tibial‐resection‐adjusted rotation‐tool flexion gaps and compartment‐specific changes.

Alignment group	Compartment	Knees, *n*	Initial manual‐stress gap (mm)	Tibial‐resection‐adjusted rotation‐tool gap (mm)	Compartment‐specific gap change (mm)
Overall	Medial	505	2.6 [1.4–4.4]	4.6 [2.8–6.2]	1.6 [−0.2 to 3.2]
	Lateral	505	5.4 [3.8–7.5]	6.5 [4.5–8.5]	1.1 [−0.8 to 2.9]
Varus	Medial	306	3.0 [1.6–4.6]	5.0 [3.3–6.4]	1.6 [−0.1 to 3.4]
	Lateral	306	5.0 [3.3–6.8]	6.0 [4.1–7.6]	1.0 [−0.8 to 2.7]
Neutral	Medial	60	2.1 [1.0–3.6]	3.8 [2.3–6.2]	1.1 [−0.4 to 3.6]
	Lateral	60	5.2 [3.7–7.9]	6.4 [4.6–8.2]	0.0 [−1.3 to 2.8]
Valgus	Medial	139	2.3 [1.2–4.0]	4.1 [2.2–5.2]	1.2 [−0.1 to 2.8]
	Lateral	139	6.5 [4.8–9.2]	8.1 [6.0–10.4]	1.5 [−0.2 to 3.2]

*Note*: Values are median [Q1–Q3]. The paired cohort included 505 knees. Adjusted rotation‐tool gaps were calculated by subtracting the corresponding robot‐validated proximal tibial resection depth from the raw distraction distance. Compartment‐specific gap change was the adjusted rotation‐tool gap minus the corresponding initial manual‐stress gap.

Abbreviations: Q1, first quartile; Q3, third quartile.

**Table 3:** Primary and secondary statistical analyses.

Analysis	Comparison or exposure	Effect estimate (95% CI)	Holm *p*
Primary compartment‐specific gap change	Medial compartment‐specific gap change	1.5 mm (1.2–1.7)	<0.001
	Lateral compartment‐specific gap change	0.8 mm (0.5–1.1)	<0.001
Alignment‐group effect	Tibial‐resection‐adjusted medial rotation‐tool gap	—	0.176
	Tibial‐resection‐adjusted lateral rotation‐tool gap	—	<0.001
Lateral alignment contrast	Valgus versus varus	1.8 mm (1.1–2.4)	<0.001
	Valgus versus neutral	1.4 mm (0.5–2.2)	0.003
Varus deformity severity	Medial compartment‐specific gap change, per 1° greater severity	0.11 mm (0.04–0.19)	0.006
	Lateral compartment‐specific gap change, per 1° greater severity	0.02 mm (−0.05 to 0.09)	0.534
	Medial gap opening ≥2 mm, per 1° greater severity	OR 1.11 (1.03–1.19)	0.012
	Lateral gap opening ≥2 mm, per 1° greater severity	OR 1.02 (0.96–1.09)	0.470
	Shift towards a larger medial gap, per 1° greater severity	0.09 mm (0.04–0.15)	0.001
Valgus deformity severity	Medial compartment‐specific gap change, per 1° greater severity	0.12 mm (0.05–0.19)	<0.001
	Lateral compartment‐specific gap change, per 1° greater severity	0.24 mm (0.14–0.34)	<0.001
	Medial gap opening ≥2 mm, per 1° greater severity	OR 1.20 (1.08–1.33)	0.001
	Lateral gap opening ≥2 mm, per 1° greater severity	OR 1.20 (1.08–1.34)	0.001
	Shift towards a larger lateral gap, per 1° greater severity	0.15 mm (0.07–0.23)	0.001
1‐year postoperative KOOS‐JR	Medial compartment‐specific gap change, per 1 mm	0.3 points (−0.2 to 0.7)	0.959
	Lateral compartment‐specific gap change, per 1 mm	0.1 points (−0.3 to 0.5)	0.959
	Mean compartment‐specific gap change, per 1 mm	0.2 points (−0.3 to 0.7)	0.959

*Note*: Primary paired effects are unadjusted mean differences. Other linear effects are *β* coefficients; logistic effects are ORs. Alignment‐group and continuous compartment‐specific severity models used the tibial‐resection‐adjusted rotation‐tool gap as the outcome; logistic models used compartment‐specific gap opening ≥2 mm. All models included the corresponding initial gap, initial flexion laxity range, age, sex and BMI. Directional models adjusted for the initial directional gap difference and the same remaining covariates. Severity was absolute preoperative HKA within each alignment group. KOOS‐JR models adjusted for preoperative KOOS‐JR, age, sex, BMI and preoperative HKA. Final implant type was excluded from these models and included only in sensitivity analyses.

Patient‐clustered HC1 standard errors and *t*‐based inference were used. CIs are pointwise. Holm correction was applied across two primary comparisons, two alignment‐group tests, three contrasts per compartment, two compartments per alignment group separately for continuous change and opening, two directional‐severity tests and three KOOS‐JR exposures. Models included 505 knees overall, 306 varus knees, 139 valgus knees and 342 knees for KOOS‐JR.

Abbreviations: BMI, body mass index; CI, confidence interval; HKA, hip–knee–ankle angle; KOOS‐JR, Knee injury and Osteoarthritis Outcome Score for Joint Replacement; OR, odds ratio.

**Figure 3:**
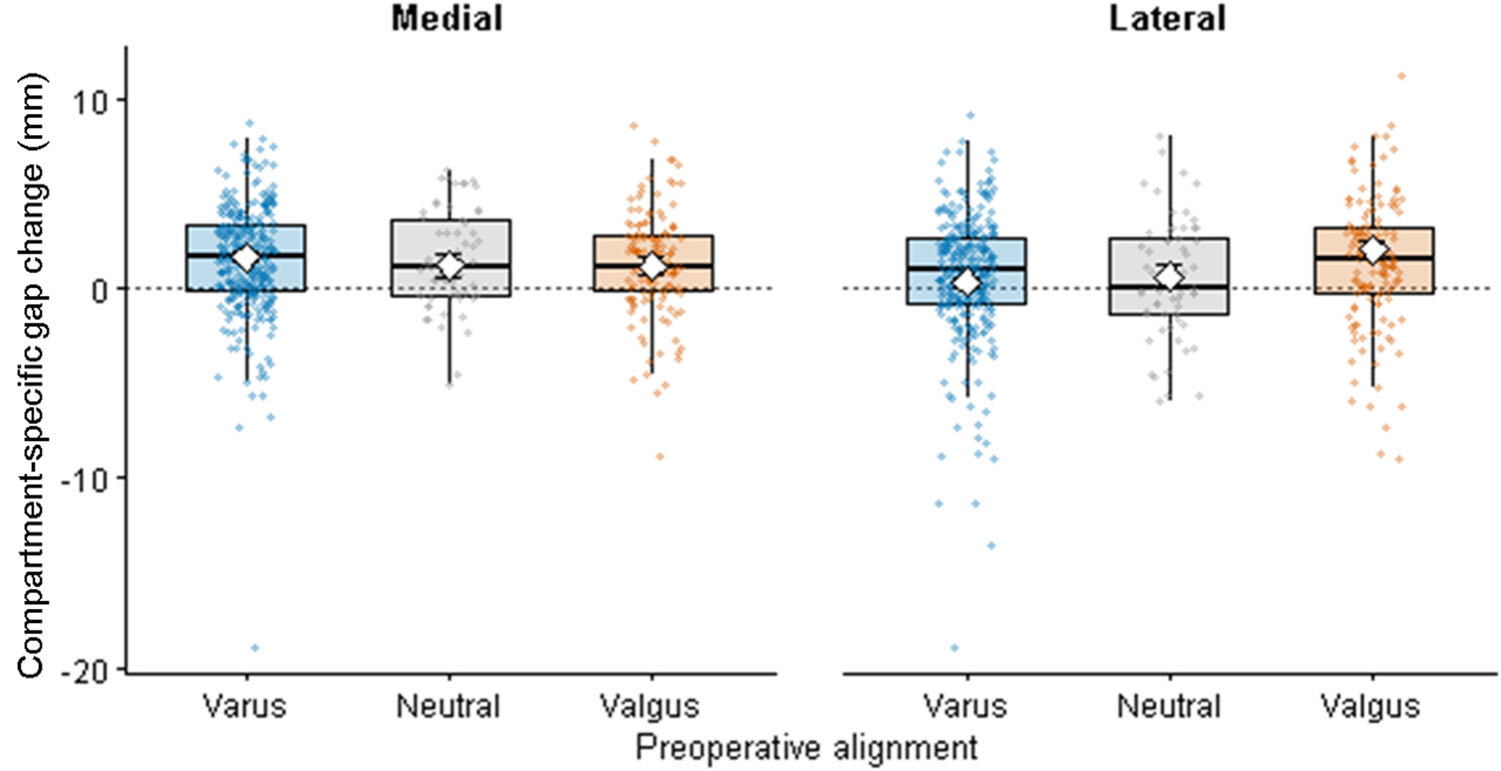
Changes in medial and lateral flexion gaps from the initial manual‐stress assessment to the post‐resection rotation‐tool assessment according to preoperative alignment. Positive values indicate larger adjusted gaps at the post‐resection rotation‐tool assessment. Boxes indicate medians and IQRs, and individual points represent knees. White diamonds and error bars indicate covariate‐adjusted mean estimates and patient‐clustered 95% CIs. Models were adjusted for the corresponding initial gap, initial flexion laxity range, age, sex and BMI. Varus, neutral and valgus groups included 306, 60 and 139 knees, respectively, and were defined as preoperative HKA ≥ 3°, −3° < HKA < 3° and HKA ≤ −3°, respectively. BMI, body mass index; CI, confidence interval; HKA, hip–knee–ankle angle; IQR, interquartile range.

Among 306 varus knees, each 1° increase in coronal deformity severity was associated with a 0.11‐mm greater medial compartment‐specific gap change (95% CI 0.04–0.19; Holm‐adjusted *p* = 0.006), whereas no association was observed in the lateral compartment (*β* = 0.02 mm, 95% CI −0.05 to 0.09; Holm‐adjusted *p* = 0.534). Among 139 valgus knees, each 1° increase in deformity severity was associated with a 0.24‐mm greater lateral gap change (95% CI 0.14–0.34; Holm‐adjusted *p* < 0.001), corresponding to approximately 1.20 mm (95% CI 0.70–1.71) per 5° greater valgus severity (Table 3; Figure 4a for the lateral‐compartment association).

**Figure 4:**
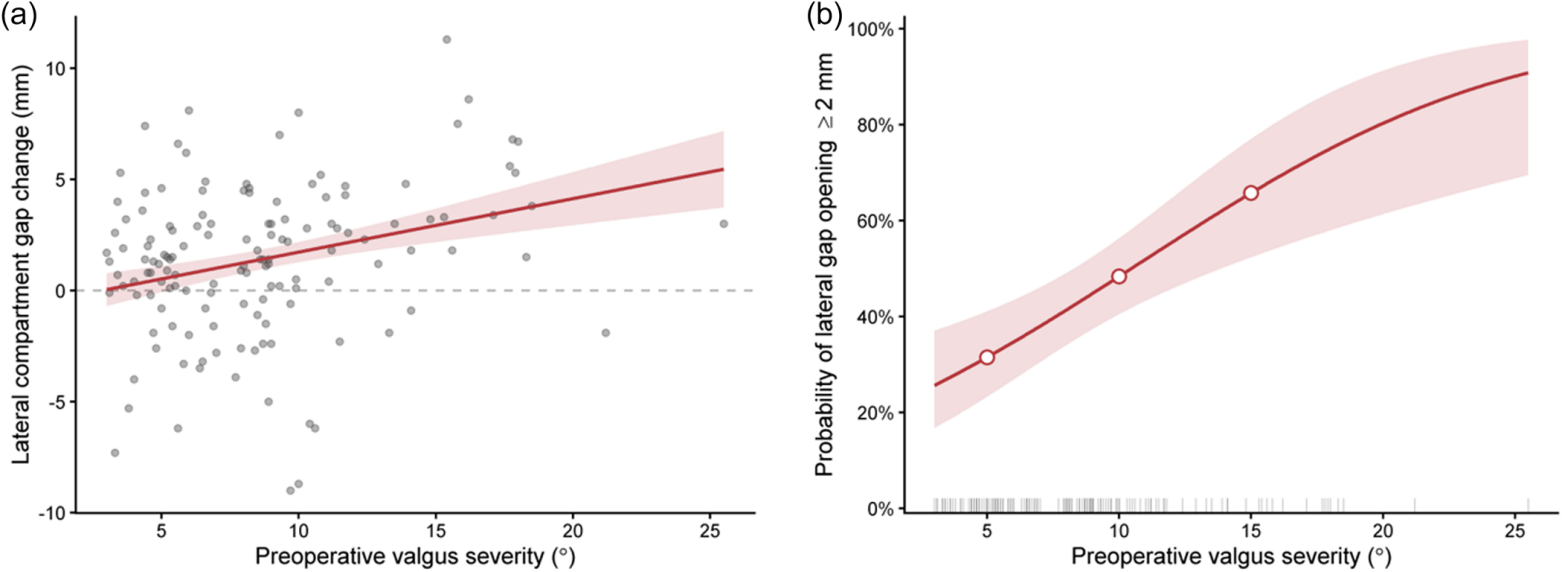
Association of preoperative valgus severity with lateral compartment gap change and lateral gap opening. (a) Relationship between preoperative valgus severity and the tibial‐resection‐adjusted rotation‐tool gap minus the initial manual‐stress gap in the lateral compartment. Points represent individual knees; the red line and shaded band indicate adjusted estimates and patient‐clustered 95% CIs from multivariable linear regression. (b) Standardized model‐estimated probability of lateral gap opening of ≥2 mm according to preoperative valgus severity. The red curve and shaded band indicate estimated probabilities and patient‐clustered 95% CIs. Open circles indicate estimates at 5°, 10° and 15° of valgus. Both models included 139 valgus knees and were adjusted for the initial lateral gap, initial flexion laxity range, age, sex and BMI. Valgus severity is expressed as positive degrees.

In varus knees, gap opening of ≥2 mm was observed medially in 143 of 306 knees (46.7%) and laterally in 109 knees (35.6%). In valgus knees, medial opening of ≥2 mm occurred in 58 of 139 knees (41.7%) and lateral opening in 60 knees (43.2%). Greater varus severity was associated with medial opening (adjusted OR per degree, 1.11; 95% CI 1.03–1.19; Holm‐adjusted *p* = 0.012), but not with lateral opening (adjusted OR per degree, 1.02; 95% CI 0.96–1.09; Holm‐adjusted *p* = 0.470). Greater valgus severity was associated with both medial opening (adjusted OR per degree, 1.20; 95% CI 1.08–1.33; Holm‐adjusted *p* = 0.001) and lateral opening (adjusted OR per degree, 1.20; 95% CI 1.08–1.34; Holm‐adjusted *p *= 0.001) (Table 3). For lateral opening of ≥2 mm in valgus knees, the model‐estimated probabilities, standardized over the covariate distribution of the model cohort, were 31.5%, 48.4% and 65.8% at 5°, 10° and 15° of valgus, respectively (Figure 4b).

### Gap balance distribution and transitions

Overall, balance increased from 44.8% (226/505) initially to 54.9% (277/505) post‐resection, an increase of 10.1 percentage points (95% CI 5.4–14.7). Imbalance with a larger lateral gap decreased from 50.1% (253/505) to 41.0% (207/505), while imbalance with a larger medial gap changed from 5.1% (26/505) to 4.2% (21/505) (Table 4).

In varus knees, balance increased from 51.3% (157/306) to 69.3% (212/306), while imbalance with a larger lateral gap decreased from 41.2% to 24.5%. Eight initially balanced varus knees (5.1%) became classified as imbalanced with a larger medial gap. In valgus knees, balance decreased from 30.2% (42/139) to 24.5% (34/139), while imbalance with a larger lateral gap increased from 69.1% to 74.8%. Of 42 initially balanced valgus knees, 21 (50.0%) were reclassified as imbalanced, including 20 (47.6%) with larger lateral gaps (Tables 4 and 5). Each degree of severity was associated with a 0.09‐mm shift towards a larger medial gap in varus knees (95% CI 0.04–0.15) and a 0.15‐mm shift towards a larger lateral gap in valgus knees (95% CI 0.07–0.23; both Holm‐adjusted *p *= 0.001). Using 2‐ and 3‐mm balance thresholds, reclassification occurred in 21/36 (58.3%) and 24/54 (44.4%) initially balanced valgus knees, respectively (Supporting Information S1: Table 3). All 21 reclassified using the 2.5‐mm threshold had final measurements: 16 (76.2%) were balanced, and 5 (23.8%) remained imbalanced.

**Table 4:** Flexion gap balance distribution across sequential intraoperative assessments.

Alignment group	Assessment	Knees, *n*	Medial gap larger, *n* (%)	Balanced, *n* (%)	Lateral gap larger, *n* (%)
Overall	Initial manual stress	505	26 (5.1)	226 (44.8)	253 (50.1)
	Post‐resection rotation tool	505	21 (4.2)	277 (54.9)	207 (41.0)
	Final trial‐component	465	6 (1.3)	370 (79.6)	89 (19.1)
Varus	Initial manual stress	306	23 (7.5)	157 (51.3)	126 (41.2)
	Post‐resection rotation tool	306	19 (6.2)	212 (69.3)	75 (24.5)
	Final trial‐component	284	3 (1.1)	230 (81.0)	51 (18.0)
Neutral	Initial manual stress	60	2 (3.3)	27 (45.0)	31 (51.7)
	Post‐resection rotation tool	60	1 (1.7)	31 (51.7)	28 (46.7)
	Final trial‐component	56	1 (1.8)	44 (78.6)	11 (19.6)
Valgus	Initial manual stress	139	1 (0.7)	42 (30.2)	96 (69.1)
	Post‐resection rotation tool	139	1 (0.7)	34 (24.5)	104 (74.8)
	Final trial‐component	125	2 (1.6)	96 (76.8)	27 (21.6)

*Note*: Balance was defined as an absolute mediolateral gap difference ≤2.5 mm. Medial gap larger and lateral gap larger denote a gap exceeding the opposite compartment by >2.5 mm. Initial and post‐resection rows use the paired cohort (505 knees); final rows use the cohort with complete measurements at all three assessments (465 knees). Figure 5 uses the same 465‐knee cohort at all three assessments.

**Table 5:** Valgus severity and lateral gap opening.

Variable	Result
Valgus knees, *n*	139
Lateral gap opening ≥2 mm, *n* (%)	60 (43.2)
Valgus severity with lateral gap opening ≥2 mm, °	9.1 [6.0–12.0]
Valgus severity with lateral gap opening <2 mm, °	6.9 [5.0–8.9]
Adjusted OR for lateral gap opening per 1° greater valgus severity	1.20 (1.08–1.34)
Holm‐adjusted *p* value	0.001
Standardized probability of lateral gap opening at 5° of valgus, % (95% CI)	31.5 (23.3–41.1)
Standardized probability of lateral gap opening at 10° of valgus, % (95% CI)	48.4 (40.5–56.3)
Standardized probability of lateral gap opening at 15° of valgus, % (95% CI)	65.8 (52.3–77.1)
Valgus knees classified as balanced at the initial assessment, *n*	42
Reclassified as imbalanced at the post‐resection assessment, *n* (%)	21 (50.0)
Post‐resection lateral gap >2.5 mm larger than the medial gap, *n* (%)	20 (47.6)
Reclassified knees with final measurements, *n*	21
Balanced at final assessment, *n* (%)	16 (76.2)

*Note*: Continuous measurements are median [Q1–Q3]. Valgus severity is expressed as positive degrees. Lateral opening was defined as a compartment‐specific gap increase ≥2 mm. The OR and standardized probabilities were estimated from the same model as Figure 4b, adjusted for the initial lateral gap, initial flexion laxity range, age, sex and BMI. Probability CIs are pointwise and patient‐clustered.

Balance was defined using a 2.5‐mm absolute mediolateral difference. Reclassification percentages use the 42 initially balanced knees; the final balanced percentage uses the 21 reclassified knees with final measurements.

Abbreviations: BMI, body mass index; CI, confidence interval; OR, odds ratio; Q1, first quartile; Q3, third quartile.

Among 465 knees with measurements available at all three assessments, 370 (79.6%) were balanced at the final trial‐component assessment. Among 125 valgus knees with complete measurements at all three assessments, 96 (76.8%) were balanced (Table 4; Figure 5).

**Figure 5:**
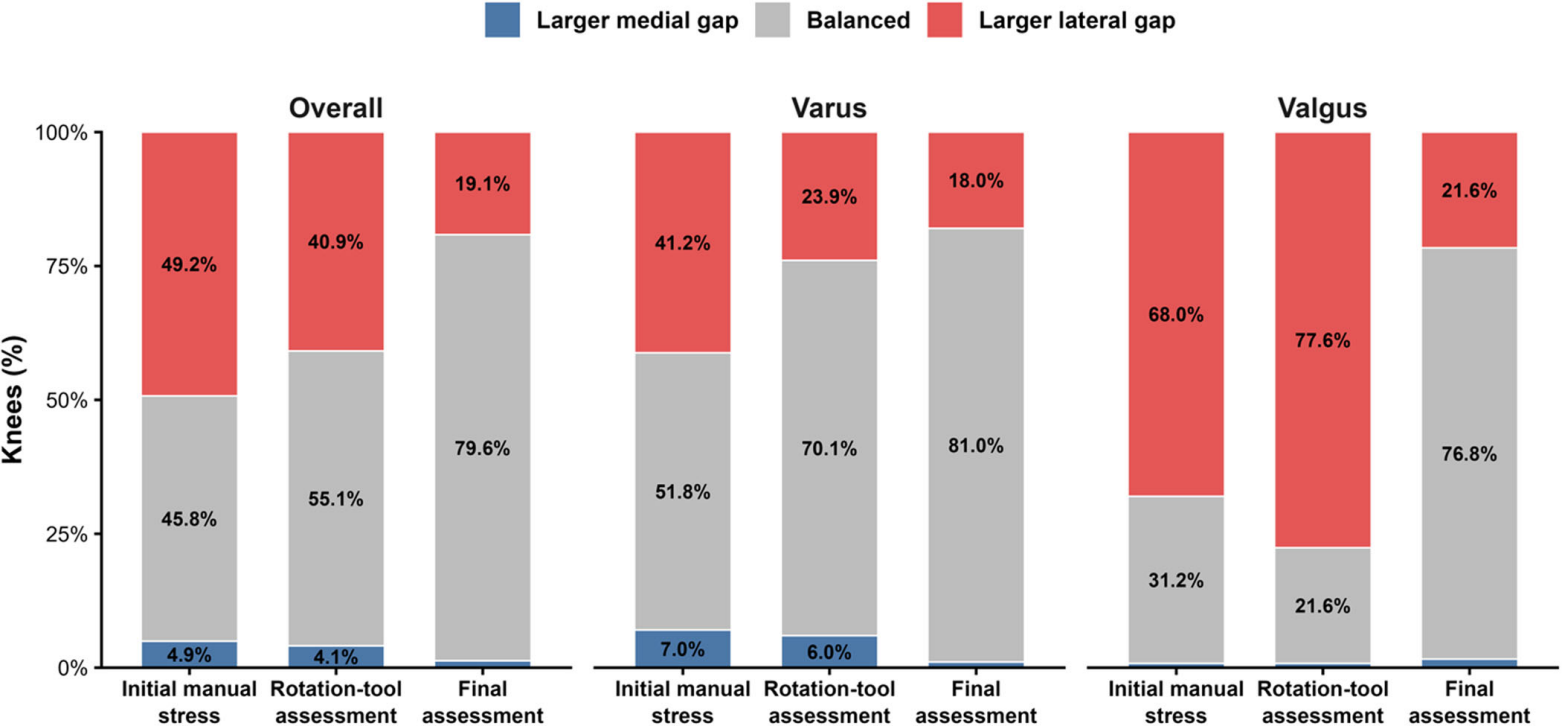
Distribution of flexion gap balance categories across three intraoperative assessments. Stacked bars show the proportions of knees with a larger medial gap, balanced gaps and a larger lateral gap at the initial manual‐stress, post‐resection rotation‐tool and final trial‐component assessments in the overall cohort and the varus and valgus subgroups. The analysis included 465 knees with complete measurements at all three assessments. Neutral knees were included in the overall cohort but are not shown separately. Balance was defined as an absolute mediolateral gap difference of ≤2.5 mm. A lateral gap more than 2.5 mm larger than the medial gap and a medial gap more than 2.5 mm larger than the lateral gap defined the two imbalance categories. Varus and valgus were defined as preoperative HKA ≥ 3° and ≤−3°, respectively. Values within bars are percentages. HKA, hip–knee–ankle angle.

Absolute change in mediolateral gap difference was not associated with final absolute difference (*β* = 0.01 mm/mm, 95% CI −0.07 to 0.08) or final imbalance overall (OR 1.04 per mm, 95% CI 0.91–1.18; both Holm‐adjusted *p* = 1.000). In exploratory valgus analyses, the association with final imbalance was nominally significant (OR 1.36 per mm, 95% CI 1.06–1.74; *p* = 0.018), but not after Holm correction across three alignment‐specific binary analyses (Holm‐adjusted *p* = 0.053; Supporting Information S1: Table 1). No adjusted associations were identified in varus or neutral knees (all Holm‐adjusted *p* ≥ 0.129).

### Secondary findings

The association between valgus severity and lateral gap change persisted in CR‐only analyses (123 knees; *β* = 0.22 mm/°, 95% CI 0.10–0.34; Holm‐adjusted *p* = 0.001) and women‐only analyses (111 knees; *β* = 0.22 mm/°, 95% CI 0.11–0.33; Holm‐adjusted *p* < 0.001). When raw rotation‐tool distraction distance was modelled with tibial resection depth as an estimated covariate, the association was attenuated but remained positive (*β* = 0.13 mm/°, 95% CI 0.02–0.24; nominal *p* = 0.022; Supporting Information S1: Table 3).

Within the 505‐knee paired cohort, preoperative KOOS‐JR was missing in 41 knees and postoperative KOOS‐JR in 150; 342 had both measurements (Supporting Information S1: Table 2). Among these 342 knees, 1‐year postoperative KOOS‐JR was not associated with medial gap change (*β* = 0.25 points/mm, 95% CI −0.24 to 0.73), lateral gap change (*β* = 0.08 points/mm, 95% CI −0.32 to 0.47) or mean gap change (*β* = 0.19 points/mm, 95% CI − 0.31 to 0.68; all Holm‐adjusted *p* = 0.959; Table 3). Additional adjustment for final implant type yielded the same conclusion (Supporting Information S1: Table 3).

## DISCUSSION

This study aimed to quantify compartment‐specific flexion‐gap changes between initial manual‐stress and post‐resection quantitative assessments and examine their relationship with coronal deformity severity. Both hypotheses were supported: gaps differed between assessments, and greater varus and valgus severity were associated with larger medial and lateral changes, respectively. Tibial‐resection‐adjusted gaps were larger in both compartments. Balance increased in varus knees, whereas half of initially balanced valgus knees were reclassified as imbalanced, almost always with larger lateral gaps. Gap changes were not associated with 1‐year KOOS‐JR.

The findings characterize two assessment conditions within the operative workflow. Pre‐resection manual stress was followed by quantitative tensioning after bone resections and extension balancing, so operative stage and tensioning method changed together. The later assessment provided gap information before femoral rotation and posterior condylar resection were finalized.

Previous L‐FKP studies have demonstrated substantial interindividual variation in flexion laxity [8, 9]. Similarly, Eller et al. reported that valgus deformity severity correlated with gap asymmetry in extension but not in flexion, with considerable variability in flexion‐gap patterns [6]. Other studies have documented changes in flexion gaps after bone resection and sequential balancing, including differences that persisted after accounting for resection depth [1, 20, 31]. The present findings add a description of compartment‐specific differences between pre‐resection manual‐stress and post‐resection quantitative assessments, including the association between greater valgus severity and larger lateral gap change.

Soft‐tissue anatomy and intervening procedures may contribute to these patterns. In varus knees, proximal tibial resection and osteophyte removal could promote medial opening; a cadaveric study showed that conventional tibial resection can involve the deep medial collateral ligament attachment [18]. In valgus knees, selective lateral release during extension balancing may contribute to subsequent lateral opening, given its effects on flexion and extension gaps [16]. These patterns occurred within different alignment strategies [10, 15]. Medial opening tended to reduce the initial lateral‐gap predominance in varus knees, whereas lateral opening accentuated it in valgus knees.

Greater valgus severity showed a continuous association with lateral opening across the observed range. Although the per‐degree effect was modest, a 5° difference corresponded to approximately 1.2 mm greater lateral gap change. This continuous pattern complements previous reports of flexion‐gap variability in valgus knees [6, 23]. Standardized probabilities of ≥2‐mm lateral opening increased from 31.5% at 5° to 65.8% at 15° of valgus, illustrating the magnitude of the severity‐related gradient within this workflow.

The transitions describe the gap information available before final femoral preparation. Initial balance frequently did not persist to the post‐resection checkpoint in valgus knees, with newly detected imbalance predominantly involving a larger lateral gap. Femoral rotation, posterior condylar resection, implant selection and final soft‐tissue balancing remained available. Subsequently, 76.8% of valgus knees with complete measurements were balanced at final trialling. Among the 21 initially balanced valgus knees reclassified as imbalanced, 16 (76.2%) were balanced at the final assessment.

Woelfle et al. reported similar femoral component planning recommendations based on pre‐resection surgeon‐applied stress and post‐resection ligament‐tensor assessment [30]. The present study complements this comparison by quantifying compartment‐specific differences and balance reclassification at the planning checkpoint. These findings describe how the gap information available for femoral planning can differ from the initial assessment.

The ≥2‐mm threshold identified substantial compartment‐specific opening between assessments. This differs from the mediolateral asymmetry used to classify balance at a single assessment. A 2‐mm tolerance is commonly used for satisfactory balance [7, 31], and differences >2 mm have been associated with inferior patient‐reported outcomes [13]. Interpretation also depends on the compartment, implant constraint and native laxity phenotype [12]. Functional knee positioning has intentionally targeted 1–2 mm greater lateral flexion laxity [28], and lateral laxity up to 6 mm has been tolerated in functionally aligned CR TKA [25]. Conversely, >4° lateral laxity in PS TKA [11] and medial laxity [24] have been associated with inferior outcomes. Thus, between‐assessment opening should be considered alongside the intended final laxity pattern.

A prospective comparison of planning based on the initial assessment alone with a workflow incorporating quantitative reassessment would help determine whether reassessment changes surgical decisions and improves final balance or patient‐reported outcomes. Such a study could also evaluate whether these effects vary with preoperative valgus severity.

Medial, lateral and mean gap changes were not associated with 1‐year KOOS‐JR in the 342 knees with available data. This negative finding is central to the clinical interpretation: larger changes in intraoperative gaps were not associated with better or worse patient‐reported outcomes at 1 year. The clinical question for future comparative studies is whether incorporating quantitative reassessment changes surgical decisions and subsequent outcomes.

Several limitations should be acknowledged. First, operative stage and tensioning method changed simultaneously, preventing separation of measurement effects from those of bone resection, deformity correction, PCL management, osteophyte removal and soft‐tissue release. Tibial‐resection subtraction corrected the geometric reference but did not recreate the initial biomechanical state. Second, the extent of posterolateral release and iliotibial band pie‐crusting was unrecorded. Their contribution could not be quantified, although release effects vary by structure and flexion angle [16, 29]. Third, varus and valgus knees underwent different alignment strategies; between‐group differences therefore reflect both anatomy and treatment. Fourth, this retrospective single‐surgeon study used one robotic platform and tensioner, limiting generalizability. Manual stress was not force‐controlled, and repeatability and measurement error were not quantified. The coefficients do not establish a minimum clinically important difference, and model‐derived probabilities are not validated treatment thresholds. Fifth, missing final‐gap and KOOS‐JR data may introduce selection bias. Alignment‐stratified final‐imbalance analyses were exploratory, and the nominal valgus association did not remain significant after multiplicity correction. Finally, there was no comparison group undergoing femoral planning based exclusively on the initial assessment. The study therefore cannot establish whether serial reassessment improves final balance or clinical outcomes.

The study's strengths include a large paired cohort, compartment‐specific adjustment for tibial resection depth and patient‐clustered analyses accounting for bilateral knees. Continuous modelling of severity further characterized gap changes beyond broad alignment categories.

## CONCLUSION

Flexion‐gap measurements and balance classifications differed between pre‐resection manual‐stress assessment and quantitative tensioning after bone resections and extension balancing. Greater valgus severity was associated with larger lateral gap changes, and half of initially balanced valgus knees were reclassified as imbalanced at the post‐resection assessment. These between‐assessment gap changes were not associated with 1‐year KOOS‐JR.

## AUTHOR CONTRIBUTIONS

**Yugo Morita**: Conceptualization; data curation; formal analysis; methodology; writing—original draft. **Jonggu Shin**: Investigation; validation; writing—review and editing. **Mohammed El‐Hassan**: Data curation; writing—review and editing. **Ittai Shichman**: Writing—review and editing. **Skylar Lewis**: Writing—review and editing. **John M. Dundon**: Writing—review and editing. **William J. Long**: Writing—review and editing. **Peter K. Sculco**: Conceptualization; project administration; resources; supervision; writing—review and editing.

## FUNDING INFORMATION

The authors have no funding to report.

## CONFLICT OF INTEREST STATEMENT

John M. Dundon reports speakers bureau and paid presentations for Zimmer Biomet; paid consultancy for Zimmer Biomet and Canary Medical; stock or stock options in Canary Medical; and research support from Zimmer Biomet and Canary Medical as a principal investigator. William J. Long reports royalties from Zimmer Biomet and Elsevier; speakers bureau and paid presentations for Zimmer Biomet, Sylke and Convatec; stock or stock options in Sylke; service on the editorial or governing board of the *Journal of Arthroplasty*; and board or committee appointments with the Hip Society, the Knee Society and the American Joint Replacement Registry Steering Committee. Peter K. Sculco reports royalties from Enovis; speakers bureau and paid presentations for Zimmer; paid consultancy for Enovis and Zimmer; stock or stock options in Intellijoint Surgical and Parvizi Surgical Innovation; research support from Intellijoint Surgical as a principal investigator; and a board or committee appointment with *HSS Journal*. The remaining authors declare no conflict of interest.

## ETHICS STATEMENT

Institutional review board approval (IRB 2025‐0624) was obtained from the participating institution. Informed consent was waived owing to the retrospective nature of the study.

## Supporting information

Supporting File 1.

## Data Availability

The data that support the findings of this study are available on request from the corresponding author. The data are not publicly available due to privacy or ethical restrictions.
