# Supplementary material for: Greater valgus severity is associated with larger lateral flexion‐gap changes between intraoperative assessments during robot‐assisted total knee arthroplasty: A retrospective cohort study
Source: J Exp Orthop. 2026 Nov 3;13(4):e70976. doi: 10.1002/jeo2.70976 (PMC13729988; doi:10.1002/jeo2.70976)
Supplement: Supplementary file 1 [file JEO2-13-e70976-s001.docx]

**Supplementary Table 1.** Association of change in mediolateral gap difference with final balance

| Alignment group | Knees, n | Final imbalance, n (%) | Final absolute gap difference, β (95% CI) | Holm *p* | Final imbalance, OR (95% CI) | Holm *p* |
| --- | --- | --- | --- | --- | --- | --- |
| Overall | 465 | 95 (20.4) | 0.01 (−0.07 to 0.08) | 1.000 | 1.04 (0.91 to 1.18) | 1.000 |
| Varus | 284 | 54 (19.0) | −0.01 (−0.11 to 0.08) | 0.755 | 0.97 (0.82 to 1.15) | 0.923 |
| Neutral | 56 | 12 (21.4) | −0.18 (−0.35 to −0.01) | 0.129 | 0.85 (0.54 to 1.33) | 0.923 |
| Valgus | 125 | 29 (23.2) | 0.13 (−0.04 to 0.30) | 0.265 | 1.36 (1.06 to 1.74) | 0.053 |

Estimates are per 1-mm greater absolute change in the signed mediolateral gap difference between the initial manual-stress and post-resection rotation-tool assessments. The signed difference was lateral minus medial gap; post-resection gaps were adjusted for the corresponding proximal tibial resection depth. Separate linear and logistic models evaluated the final absolute mediolateral gap difference and final imbalance (>2.5 mm), respectively. Analyses included 465 knees with complete measurements at all three assessments. Models were adjusted for initial absolute mediolateral gap difference, initial flexion laxity range, and preoperative HKA, with patient-clustered HC1 standard errors.

All p values shown in the table are Holm-adjusted: across the two outcomes for the overall cohort and across the three alignment groups separately for each outcome. For the valgus binary model, the nominal p value was 0.018, the Holm-adjusted p value across the three alignment-specific binary analyses was 0.053, and the Holm-adjusted p value across all six alignment-specific tests was 0.106. These analyses were exploratory. Confidence intervals are pointwise and were not adjusted for multiplicity.

CI, confidence interval; HKA, hip–knee–ankle angle; OR, odds ratio.

**Supplementary Table 2.** Characteristics according to availability of paired measurements, final measurements and KOOS-JR

| Characteristic | Paired gap measurements | | | Final gap measurements | | | | Paired KOOS-JR | | | |
| --- | --- | --- | --- | --- | --- | --- | --- | --- | --- | --- | --- |
|  | Available (n = 505) | Missing (n = 7) | SMD | Available (n = 465) | Missing (n = 40) | | SMD | Available (n = 342) | Missing (n = 163) | | SMD |
| Age, years | 70.0  [64.0 to 76.0] | 77.0  [73.0 to 80.5] | 1.06 | 70.0  [64.0 to 76.0] | | 70.0  [62.5 to 76.8] | 0.08 | 70.0  [64.0 to 76.0] | | 70.0  [64.0 to 77.0] | 0.09 |
| Female sex, n (%) | 315 (62.4) | 5 (71.4) | 0.19 | 289 (62.2) | | 26 (65.0) | 0.06 | 210 (61.4) | | 105 (64.4) | 0.06 |
| BMI, kg/m² | 31.2  [27.1 to 34.4] | 25.7  [25.2 to 28.6] | 0.77 | 31.2  [27.2 to 34.3] | | 31.6  [27.1 to 35.1] | 0.00 | 31.2  [27.3 to 34.5] | | 31.1  [27.1 to 34.2] | 0.06 |
| Preoperative HKA, ° | 6.0  [−4.0 to 10.5] | 8.4  [5.4 to 10.8] | 0.65 | 6.1  [−3.6 to 10.5] | | 5.0  [−5.7 to 9.7] | 0.21 | 5.5  [−4.6 to 10.3] | | 6.8  [−0.7 to 11.3] | 0.17 |
| Neutral alignment, n (%) | 60 (11.9) | 1 (14.3) | 0.07 | 56 (12.0) | | 4 (10.0) | 0.07 | 38 (11.1) | | 22 (13.5) | 0.07 |
| Valgus alignment, n (%) | 139 (27.5) | 0 (0.0) | 0.87 | 125 (26.9) | | 14 (35.0) | 0.18 | 106 (31.0) | | 33 (20.2) | 0.25 |
| Varus alignment, n (%) | 306 (60.6) | 6 (85.7) | 0.59 | 284 (61.1) | | 22 (55.0) | 0.12 | 198 (57.9) | | 108 (66.3) | 0.17 |
| Preoperative MPTA, ° | 86.8  [84.4 to 89.3] | 85.1  [81.8 to 87.2] | 0.65 | 86.8  [84.4 to 89.0] | | 87.3  [84.8 to 90.6] | 0.26 | 86.9  [84.4 to 89.5] | | 86.7  [84.5 to 88.7] | 0.07 |
| Preoperative LDFA, ° | 87.8  [85.5 to 90.2] | 87.4  [86.8 to 88.7] | 0.15 | 87.9  [85.6 to 90.3] | | 87.4  [85.4 to 89.6] | 0.15 | 87.7  [85.4 to 90.2] | | 88.4  [86.2 to 90.2] | 0.12 |
| Preoperative JLCA, ° | 3.6  [−0.9 to 5.8] | 4.5  [3.6 to 5.8] | 0.65 | 3.6  [−0.7 to 5.8] | | 3.5  [−2.7 to 5.5] | 0.16 | 3.5  [−1.0 to 5.6] | | 4.0  [0.1 to 6.2] | 0.20 |
| CR implant, n (%) | 451 (89.3) | 5 (71.4) | 0.46 | 416 (89.5) | | 35 (87.5) | 0.06 | 311 (90.9) | | 140 (85.9) | 0.16 |
| PS implant, n (%) | 54 (10.7) | 2 (28.6) | 0.46 | 49 (10.5) | | 5 (12.5) | 0.06 | 31 (9.1) | | 23 (14.1) | 0.16 |
| Initial medial gap, mm | 2.6 [1.4 to 4.4] | 2.4 [1.1 to 4.2] | 0.08 | 2.7 [1.5 to 4.4] | | 2.4 [1.0 to 4.4] | 0.03 | 2.6 [1.5 to 4.5] | | 2.8 [1.3 to 4.3] | 0.09 |
| Initial lateral gap, mm | 5.4 [3.8 to 7.5] | 4.4 [4.0 to 4.5] | 0.14 | 5.4 [3.6 to 7.5] | | 6.2 [4.5 to 7.8] | 0.09 | 5.5 [3.9 to 7.7] | | 5.3 [3.5 to 7.2] | 0.11 |
| Initial flexion laxity range, ° | 10.5  [3.6 to 14.4] | 3.3  [1.6 to 5.3] | 0.93 | 10.6  [3.8 to 14.4] | | 8.7 [1.8 to 14.9] | 0.08 | 11.0  [6.0 to 14.7] | | 8.7  [2.8 to 13.4] | 0.30 |
| Preoperative KOOS-JR | 52.5  [42.3 to 59.9] (n = 464) | 43.6  [36.2 to 44.9] (n = 6) | 0.76 | 52.5  [42.3 to 59.4] (n = 430) | | 52.4 [35.6 to 61.6] (n = 34) | 0.10 | 52.5  [43.0 to 59.4] | | 52.5  [39.6 to 61.6] (n = 122) | 0.09 |
| Medial gap change, mm | — | — | — | 1.5  [−0.2 to 3.2] | | 1.9  [0.4 to 4.3] | 0.22 | 1.2  [−0.3 to 3.2] | | 1.9  [0.3 to 3.5] | 0.24 |
| Lateral gap change, mm | — | — | — | 1.0  [−0.8 to 2.8] | | 1.4  [−0.4 to 4.2] | 0.29 | 1.1  [−0.8 to 2.9] | | 1.1  [−0.8 to 3.0] | 0.07 |

Values are median [Q1 to Q3] or n (%). Final-gap and KOOS-JR comparisons are within the 505-knee paired cohort. Available n is stated when lower than the column denominator. SMDs are absolute standardised mean differences, based on means/standard deviations or proportions. Comparisons are descriptive; no hypothesis tests were performed. Gap changes require paired measurements and are therefore not shown for the first comparison.

BMI, body mass index; CR, cruciate-retaining; HKA, hip–knee–ankle angle; JLCA, joint-line convergence angle; KOOS-JR, Knee injury and Osteoarthritis Outcome Score for Joint Replacement; LDFA, lateral distal femoral angle; MPTA, medial proximal tibial angle; PS, posterior-stabilised; Q1/Q3, first/third quartiles.

**Supplementary Table 3.** Sensitivity analyses of gap change, compartment opening and clinical outcomes

| Analysis | Group | Side | n (events) | Effect estimate (95% CI) | *p* |
| --- | --- | --- | --- | --- | --- |
| Implant-adjusted: gap change | Varus | Medial | 306 | β 0.11 (0.04 to 0.19) | 0.006 |
| Implant-adjusted: gap change | Varus | Lateral | 306 | β 0.02 (−0.06 to 0.09) | 0.642 |
| Implant-adjusted: gap change | Valgus | Medial | 139 | β 0.09 (0.02 to 0.16) | 0.009 |
| Implant-adjusted: gap change | Valgus | Lateral | 139 | β 0.22 (0.12 to 0.33) | <0.001 |
| CR only: gap change | Varus | Medial | 274 | β 0.14 (0.07 to 0.20) | <0.001 |
| CR only: gap change | Varus | Lateral | 274 | β 0.04 (−0.04 to 0.11) | 0.299 |
| CR only: gap change | Valgus | Medial | 123 | β 0.09 (0.02 to 0.16) | 0.018 |
| CR only: gap change | Valgus | Lateral | 123 | β 0.22 (0.10 to 0.34) | 0.001 |
| Women only: gap change | Varus | Medial | 166 | β 0.09 (0.00 to 0.18) | 0.091 |
| Women only: gap change | Varus | Lateral | 166 | β −0.02 (−0.12 to 0.08) | 0.676 |
| Women only: gap change | Valgus | Medial | 111 | β 0.10 (0.03 to 0.16) | 0.006 |
| Women only: gap change | Valgus | Lateral | 111 | β 0.22 (0.11 to 0.33) | <0.001 |
| Implant-adjusted: opening ≥2 mm | Varus | Medial | 306 (143) | OR 1.11 (1.03 to 1.19) | 0.015 |
| Implant-adjusted: opening ≥2 mm | Varus | Lateral | 306 (109) | OR 1.02 (0.96 to 1.09) | 0.515 |
| Implant-adjusted: opening ≥2 mm | Valgus | Medial | 139 (58) | OR 1.17 (1.05 to 1.30) | 0.004 |
| Implant-adjusted: opening ≥2 mm | Valgus | Lateral | 139 (60) | OR 1.19 (1.07 to 1.32) | 0.004 |
| CR only: opening ≥2 mm | Varus | Medial | 274 (124) | OR 1.11 (1.02 to 1.20) | 0.028 |
| CR only: opening ≥2 mm | Varus | Lateral | 274 (95) | OR 1.03 (0.96 to 1.11) | 0.386 |
| CR only: opening ≥2 mm | Valgus | Medial | 123 (47) | OR 1.16 (1.04 to 1.29) | 0.007 |
| CR only: opening ≥2 mm | Valgus | Lateral | 123 (49) | OR 1.19 (1.06 to 1.34) | 0.006 |
| Women only: opening ≥2 mm | Varus | Medial | 166 (73) | OR 1.04 (0.94 to 1.16) | 0.869 |
| Women only: opening ≥2 mm | Varus | Lateral | 166 (57) | OR 0.97 (0.88 to 1.06) | 0.869 |
| Women only: opening ≥2 mm | Valgus | Medial | 111 (51) | OR 1.19 (1.06 to 1.33) | 0.006 |
| Women only: opening ≥2 mm | Valgus | Lateral | 111 (50) | OR 1.19 (1.06 to 1.32) | 0.006 |
| Opening ≥1 mm | Varus | Medial | 306 (178) | OR 1.12 (1.04 to 1.21) | 0.004 |
| Opening ≥1 mm | Varus | Lateral | 306 (153) | OR 1.03 (0.96 to 1.10) | 0.393 |
| Opening ≥1 mm | Valgus | Medial | 139 (79) | OR 1.12 (1.01 to 1.24) | 0.025 |
| Opening ≥1 mm | Valgus | Lateral | 139 (82) | OR 1.20 (1.08 to 1.33) | 0.002 |
| Opening ≥3 mm | Varus | Medial | 306 (99) | OR 1.14 (1.06 to 1.23) | <0.001 |
| Opening ≥3 mm | Varus | Lateral | 306 (66) | OR 1.04 (0.95 to 1.13) | 0.397 |
| Opening ≥3 mm | Valgus | Medial | 139 (31) | OR 1.13 (1.03 to 1.24) | 0.013 |
| Opening ≥3 mm | Valgus | Lateral | 139 (44) | OR 1.27 (1.13 to 1.44) | <0.001 |
| Raw-gap model with resection depth | Varus | Medial | 306 | β 0.01 (−0.05 to 0.07) | 0.798† |
| Raw-gap model with resection depth | Varus | Lateral | 306 | β 0.02 (−0.05 to 0.09) | 0.575† |
| Raw-gap model with resection depth | Valgus | Medial | 139 | β 0.08 (0.01 to 0.15) | 0.021† |
| Raw-gap model with resection depth | Valgus | Lateral | 139 | β 0.13 (0.02 to 0.24) | 0.022† |
| Final imbalance >2 mm | Overall | — | 465 (121) | OR 1.03 (0.91 to 1.17) | 0.592† |
| Final imbalance >2 mm | Varus | — | 284 (71) | OR 0.98 (0.83 to 1.16) | 0.828† |
| Final imbalance >2 mm | Neutral | — | 56 (13) | OR 0.79 (0.49 to 1.27) | 0.318† |
| Final imbalance >2 mm | Valgus | — | 125 (37) | OR 1.31 (1.04 to 1.66) | 0.021† |
| Final imbalance >3 mm | Overall | — | 465 (68) | OR 1.02 (0.88 to 1.18) | 0.755† |
| Final imbalance >3 mm | Varus | — | 284 (35) | OR 1.00 (0.82 to 1.23) | 0.973† |
| Final imbalance >3 mm | Neutral | — | 56 (9) | OR 0.76 (0.45 to 1.26) | 0.277† |
| Final imbalance >3 mm | Valgus | — | 125 (24) | OR 1.21 (0.93 to 1.58) | 0.147† |
| Implant-adjusted KOOS-JR | Overall | Medial | 342 | β 0.26 (−0.23 to 0.74) | 0.901 |
| Implant-adjusted KOOS-JR | Overall | Lateral | 342 | β 0.09 (−0.31 to 0.49) | 0.901 |
| Implant-adjusted KOOS-JR | Overall | Mean | 342 | β 0.20 (−0.29 to 0.69) | 0.901 |

n is the number of knees; event counts are given for binary outcomes. †Nominal p; other p values are Holm-adjusted across two compartments within each alignment group and analysis variant/threshold, or across three KOOS-JR exposures. Patient-clustered HC1 standard errors were used; CIs are pointwise. The corresponding main-model results appear in Table 3 and Supplementary Table 1.

Severity effects are per 1° greater absolute HKA (β in mm; OR for opening). These models adjusted for the corresponding initial gap, initial flexion laxity range, age, sex and BMI; sex was omitted for women-only analyses. Implant-adjusted models additionally included final implant type. Raw-gap models used raw distraction distance as the outcome and included tibial resection depth as an estimated covariate.

Final-imbalance ORs are per 1-mm greater absolute change in the signed mediolateral gap difference, adjusted as in Supplementary Table 1. KOOS-JR β values are points per 1-mm gap change, adjusted for preoperative KOOS-JR, age, sex, BMI, HKA and implant type.

With a 2-mm balance threshold, 21/36 initially balanced valgus knees (58.3%) were reclassified as imbalanced; 13/20 with final measurements (65.0%) were finally balanced and 7/20 (35.0%) remained imbalanced. With a 3-mm threshold, the corresponding figures were 24/54 (44.4%), 20/24 (83.3%) and 4/24 (16.7%). The principal 2.5-mm results are reported in Table 5. CR, cruciate-retaining; OR, odds ratio.
